# Investigation of allele-specific expression of genes involved in adipogenesis and lipid metabolism suggests complex regulatory mechanisms of *PPARGC1A* expression in porcine fat tissues

**DOI:** 10.1186/s12863-018-0696-6

**Published:** 2018-11-29

**Authors:** Monika Stachowiak, Izabela Szczerbal, Krzysztof Flisikowski

**Affiliations:** 1 Department of Genetics and Animal Breeding, Poznan University of Life Sciences, Wolynska 33, 60-637 Poznan, Poland; 2 Chair of Livestock Biotechnology, Technical University of Munich, Liesel-Beckmannstr. 1, 85354 Freising, Germany

**Keywords:** Allele-specific expression, CpG methylation, Fat, Muscle, Pig, *PPARA*, *PPARG*, *PPARGC1A*, *SREBF1*

## Abstract

**Background:**

The expression of genes involved in regulating adipogenesis and lipid metabolism may affect economically important fatness traits in pigs. Allele-specific expression (ASE) reflects imbalance between allelic transcript levels and can be used to identify underlying *cis-*regulatory elements. ASE has not yet been intensively studied in pigs. The aim of this investigation was to analyze the differential allelic expression of four genes, *PPARA, PPARG, SREBF1,* and *PPARGC1A,* which are involved in the regulation of fat deposition in porcine subcutaneous and visceral fat and *longissimus dorsi* muscle.

**Results:**

Quantification of allelic proportions by pyrosequencing revealed that both alleles of *PPARG* and *SREBF1* are expressed at similar levels. *PPARGC1A* showed the greatest ASE imbalance in fat deposits in Polish Large White (PLW), Polish Landrace and Pietrain pigs; and *PPARA* in PLW pigs. Significant deviations of mean *PPARGC1A* allelic transcript ratio between cDNA and genomic DNA were detected in all tissues, with the most pronounced difference (*p* < 0.001) in visceral fat of PLW pigs. To search for potential *cis*-regulatory elements affecting ASE in the *PPARGC1A* gene we analyzed the effects of four SNPs (rs337351686, rs340650517, rs336405906 and rs345224049) in the promoter region, but none were associated with ASE in the breeds studied. DNA methylation analysis revealed significant CpG methylation differences between samples showing balanced (allelic transcript ratio ≈1) and imbalanced allelic expression for CpG site at the genomic position in chromosome 8 (SSC8): 18527678 in visceral fat (*p* = 0.017) and two CpG sites (SSC8:18525215, *p* = 0.030; SSC8:18525237, *p* = 0.031) in subcutaneous fat.

**Conclusions:**

Our analysis of differential allelic expression suggests that *PPARGC1A* is subjected to *cis*-regulation in porcine fat tissues. Further studies are necessary to identify other regulatory elements localized outside the *PPARGC1A* proximal promoter region.

**Electronic supplementary material:**

The online version of this article (10.1186/s12863-018-0696-6) contains supplementary material, which is available to authorized users.

## Background

Fat deposition in pigs is a complex trait that is substantially influenced by multiple genetic factors, including regulatory mutations and epimutations affecting gene expression [1, 2]. Gaining an understanding of factors involved in the regulation of lipid accumulation and fatty acid synthesis is important because excessive fatness has a negative effect on breeding efficiency. Moreover, consumers have become more interested in lean meat with high nutritional properties, and value the sensory attributes that are often influenced by fat content [3].

Allele-specific expression (ASE) contributes to the complexity of the transcriptome and reflects imbalance of expression between parental alleles. In contrast to random (e.g. X-chromosome inactivation), or non-random (imprinting) monoallelic expression where one allele is completely silenced, ASE is associated with more subtle differences in transcript level [4, 5]. This can be a consequence of variations in *cis*-regulatory DNA regions involved in transcription efficiency or transcript stability, allele-specific DNA methylation, allele-specific histone modification or location of the chromosomal territory within the nucleus [6, 7]. It is generally assumed that *trans*-regulatory elements do not contribute to ASE because both alleles are exposed to the same environmental factors. The frequency of ASE varies with species, tissue and individual physiological status, and analysis of its pattern may elucidate underlying regulatory mechanisms affecting many complex traits [8, 9]. About 20% of human genes are estimated to preferentially express one allele [10, 11], and recent high throughput RNA sequencing studies suggest it may be even more widespread [12]. For example, 52% of genes exhibit ASE in pig brain [13], and 89% of bovine genes showed allelic imbalance in at least one of 18 tissues tested in a single individual [14]. Where the same allele is over-represented in unrelated heterozygous individuals (one-directional ASE), this suggests predominant regulation by *cis*-elements closely linked to the gene. In contrast, cases of ASE that show no such consistency (bi-directional ASE) suggest regulatory elements not in a strong linkage disequilibrium with the gene [15].

Allele-specific expression of genes encoding transcriptional regulators of adipogenesis and lipid metabolism may contribute to phenotypic variation of pig fatness traits because even subtle differences may affect their expression. Moreover, ASE can be used to explore the role of putative *cis*-regulatory factors. Here we investigated the allelic expression of *PPARA* (peroxisome proliferator activated receptor alpha), *PPARG* (peroxisome proliferator activated receptor gamma), *PPARGC1A* (*PPARG* coactivator 1 alpha) and *SREBF1* (sterol regulatory element binding transcription factor 1) in subcutaneous and visceral fat deposits and in the skeletal *longissimus dorsi* (*l. dorsi*) muscle of several pig breeds. We then investigated several potential *cis*-regulatory elements including genetic variants and DNA methylation in promoter regions for their possible role in tissue-specific ASE.

## Results

### Genotyping and analysis of allele-specific expression

We first identified animals heterozygous for an exonic reporter SNP (rSNP) in each gene to enable quantification of expression of each allele in transcript pools. One hundred forty-five pigs were genotyped (Additional file 1), and at least 10 heterozygotes found in Polish Large White (PLW) and Duroc for *PPARA* (rSNP: rs342258309, A > G); PLW, Polish Landrace (PL) and Duroc for *PPARG* (rSNP: rs319172675, A > G); PLW, PL, Duroc and Pietrain for *PPARGC1A* (rSNP: rs45430917, A > T); and PL, Duroc and Pietrain breed for *SREBF1* (rSNP: rs712230598, C > T). Allelic transcript proportions were determined by pyrosequencing of cDNA reverse transcribed from RNA extracted from subcutaneous fat, visceral fat and *l. dorsi* muscle. Individual samples were classified as showing ASE when the allelic transcript ratio (percentage of one allele divided by the other) was < 0.667 or > 1.5, corresponding to allelic proportions greater than 40:60 or 60:40.

*PPARG* and *SREBF1* showed no evidence of ASE in any sample (Additional file 2). Analysis of *PPARA* revealed ASE with preferential expression of allele A in two samples (20%) of subcutaneous fat and one sample (10%) of visceral fat from the PLW breed. The greatest allelic imbalance (allelic transcript ratio = 2.23) was detected in the one sample in visceral fat (Additional file 2). Comparison of mean log_10_-transformed *PPARG*, *SREBF1* and *PPARA* allelic transcript ratio between cDNA and gDNA in the breeds tested did not reveal statistically significant differences in the tissues studied (data not shown).

**Fig. 1:**
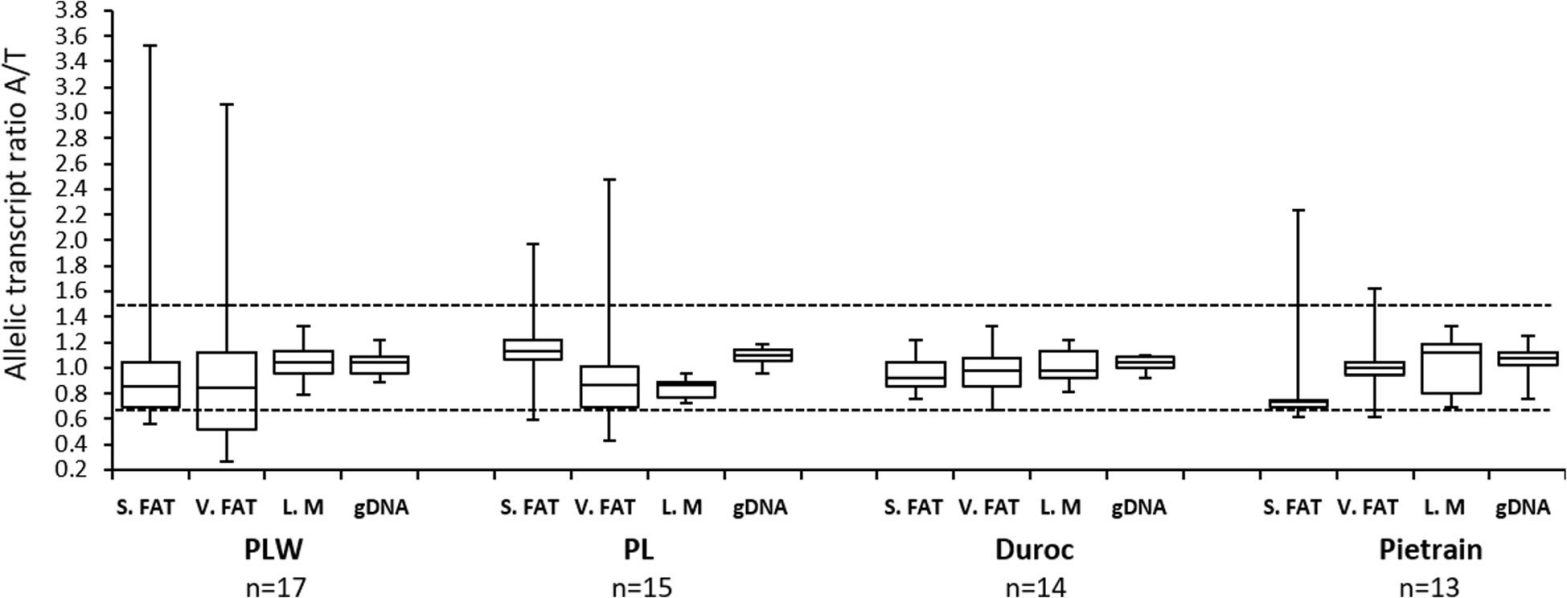
Distribution of allelic transcript ratios for *PPARGC1A* in tissues and genomic DNA of analyzed breeds. Each boxplot shows the first quartile, median, third quartile and the whiskers show the minimum and maximum allelic transcript ratio values. S. FAT – subcutaneous fat, V. FAT – visceral fat, L. M. – *longissimus dorsi* muscle, gDNA – genomic DNA

**Fig. 2:**
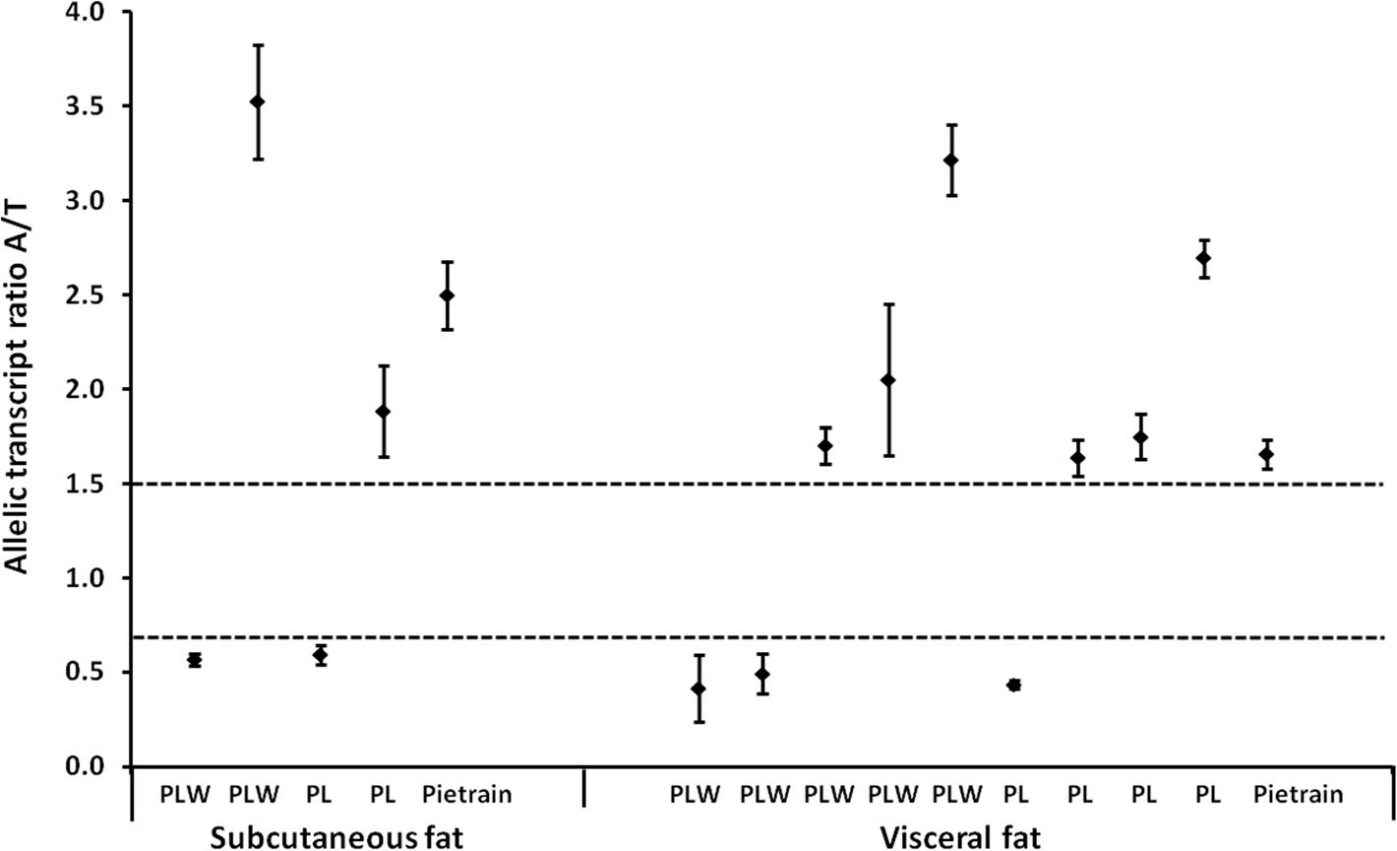
Allelic transcript ratios (mean of two measurements) ± SD of individual samples showing ASE of *PPARGC1A* in subcutaneous and visceral fat. Threshold allelic transcript ratio values (0.667 and 1.5) are marked with a dashed line

Analysis of *PPARGC1A* revealed markedly disproportionate allelic expression in individual samples of subcutaneous and visceral fat from PL, PLW and Pietrain pigs (Fig. 1). The nature of ASE was bi-directional, and the A/T allelic transcript ratios varied between 0.57 and 3.52 in subcutaneous fat, and 0.41 and 3.22 in visceral fat (Fig. 2). ASE was more common in visceral fat of PLW (29%) and PL pigs (27%) than in subcutaneous fat (12 and 13%, respectively). ASE was rare in Pietrain pigs (8%) in both fat deposits. Analysis of mean log_10_-transformed allelic transcript ratios between cDNA and genomic DNA revealed significant deviations in subcutaneous fat of PLW, PL and Duroc pigs, in visceral fat of all breeds and in *l. dorsi* muscle of PL and Duroc pigs (Table 1). The most significant differences (*p* < 0.001) were observed in visceral fat of PLW pigs.

**Table 1:** Mean log_10_-transformed *PPARGC1A* allelic transcript ratios in cDNA derived from subcutaneous fat, visceral fat and *l. dorsi* muscle, and genomic DNA (gDNA). Data were calculated after neutralizing bi-directional nature of *PPARGC1A* allelic expression

Breed^a^	Subcutaneous fat^b^	Visceral fat^b^	*L. dorsi* muscle^b^	gDNA
PLW	0.149 ^**^	0.176^***^	0.046	0.032
PL	0.072^*^	0.130^**^	0.054^**^	0.021
Duroc	0.052^*^	0.070^**^	0.047^**^	0.022
Pietrain	0.077	0.094^*^	0.041	0.040

^a^*PLW* Polish Large White, *PL* Polish Landrace

^b^Significant differences between cDNA and gDNA are shown at *p* < 0.05 (^*^), *p* < 0.01 (^**^) and *p* < 0.001 (^***^)

These data led us investigate potential *cis*-regulatory factors that may affect *PPARGC1A* expression in porcine tissues.

### Potential regulatory variants in the *PPARGC1A* 5′-flanking region

A ~ 1 kb region upstream of the translation initiation site, including the entire 5’UTR (5′-untranslated region) and the proximal promoter was sequenced to search for DNA polymorphisms. Four SNPs were identified in the promoter: rs331429264 (c.-393G > C), rs337351686 (c.-530G > A), rs318575008 (c.-531C > G) and rs340650517 (c.-644G > A), but none in the 5’UTR. We also genotyped the rs336405906 (c.-2885G > T) and rs345224049 (c.-2894G > A) SNPs in the distal promoter region to verify their effects on *PPARGC1A* mRNA expression previously reported by Kim et al. [16]. In silico analysis predicted that three of the SNPs disrupt putative consensus sites for transcription factors expressed in adipose tissue and/or skeletal muscle (Additional file 3). Of these, the ATF2 binding sequence was disturbed by rs331429264, that of STAT5A and STAT5B by rs337351686, and KLF4 and TP53 by rs340650517. These transcription factors are known regulators of adipogenesis, adipocyte function and have been implicated in human obesity and fatness traits in animals (Additional file 3). ATF2 and TP53 have been reported to regulate *PPARGC1A* promoter activity [17, 18]. To examine the association between promoter SNPs and *PPARGC1A* mRNA expression, we compared allelic transcript ratios derived from animals carrying different genotypes. This approach reduces possible confounding effects of *trans*-regulatory and environmental factors on mRNA expression because transcript abundance is compared within the same sample, not between samples [19]. Due to sufficient genotype distributions (Additional file 4), we could apply this method to test regulatory effects of rs340650517, rs337351686 and rs336405906 in PLW, and rs345224049 in PL pigs. We found that rs340650517, rs337351686 and rs336405906 SNPs segregated in PLW pigs as two haplotypes [G;G;G] and [A;A;T] and were thus analyzed together. The comparison of mean log_10_-transformed allelic transcript ratios between [G;G;G]/[G;G;G] and [G;G;G]/[A;A;T] diplotype groups revealed no significant effect on allelic transcript ratio in subcutaneous (*p* = 0.25) or visceral fat (*p* = 0.83), or *l. dorsi* muscle (*p* = 0.25). Similarly, rs345224049 SNP did not significantly affect the allelic transcript ratio in subcutaneous (*p* = 0.22) or visceral fat deposits (*p* = 0.37), or *l. dorsi* muscle (*p* = 0.08). We thus conclude that the observed ASE of *PPARGC1A* did not result from regulatory effects of rs337351686, rs340650517, rs336405906 and rs345224049 SNPs in the analyzed samples.

### CpG methylation analysis in the *PPARGC1A* region

**Fig. 3:**
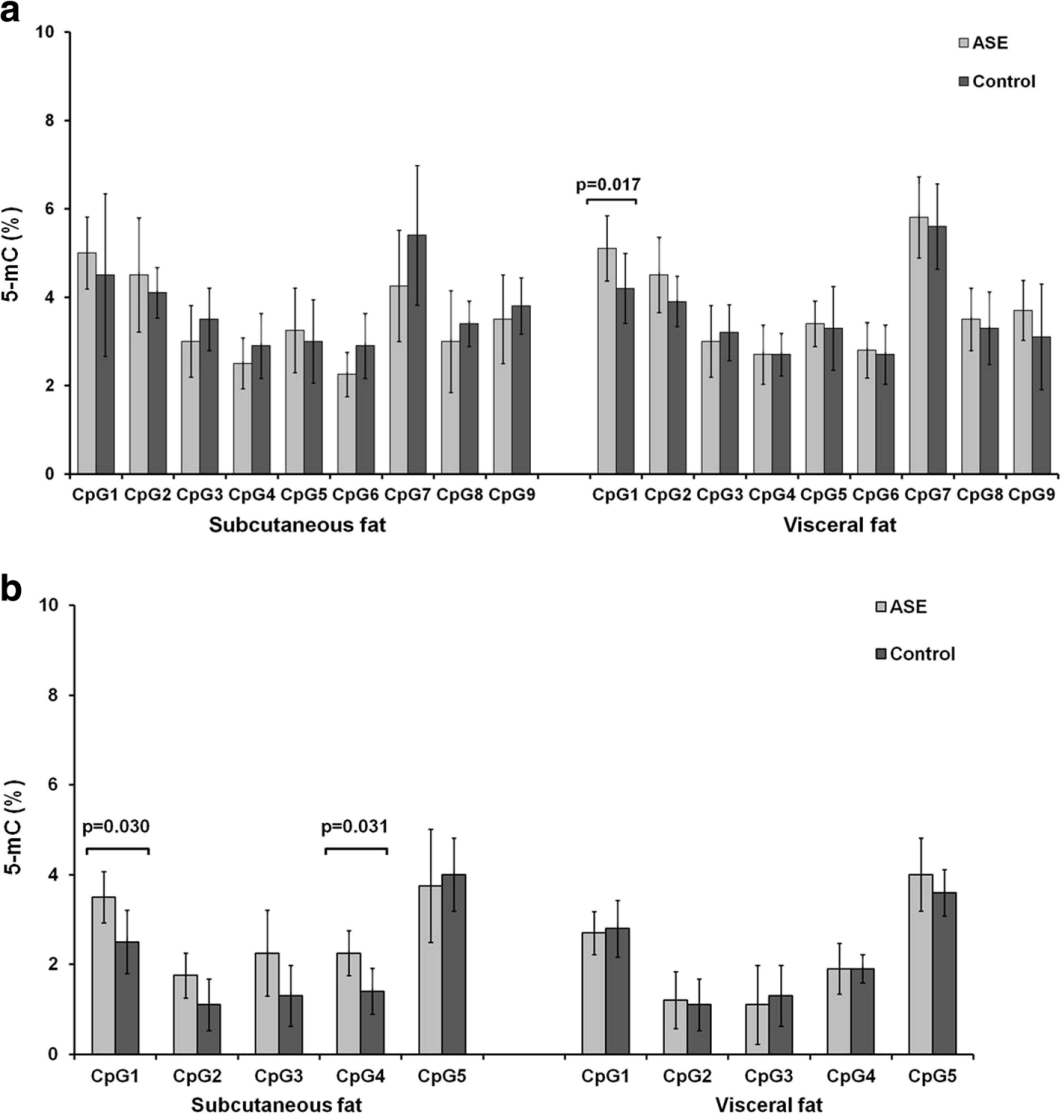
Mean percentage of 5-methylcytosine (5-mC) ± SD within CpG islands located in 5′-flanking region of *PPARGC1A* in fat deposits. The particular cytosines in each fragment analyzed: CGi1 (**a**) and CGi2 (**b**), are indicated as CpG1, CpG2, etc. *P* value is shown for cytosines that differed significantly in methylation level between ASE samples (*n* = 5 for subcutaneous fat and *n* = 10 for visceral fat) and control groups (*n* = 10 for subcutaneous and *n* = 10 for visceral fat) with similar expression of both alleles

A search for epigenetic regulatory elements that may affect expression of *PPARGC1A* transcripts via CpG methylation was performed at three CpG islands (CGi) selected based on porcine genome data (Sscrofa11.1) for chromosome 8 (SSC8). CGi1 and CGi2 are located in the 5′-flanking region (genomic positions SSC8:18527230–18528335 and 18524520–18526314, respectively) and CGi3 in exon 6 of *PPARGC1A* (SSC8:17866933–17867385). We compared 5-methylcytosine (5-mC) levels (%) in subcutaneous and visceral fat tissues between samples displaying ASE (allelic transcript ratio exceeding 0.667–1.5 range) and control samples with similar expression of both alleles (mean allelic transcript ratio ± SD was 0.98 ± 0.09 in subcutaneous fat and 1.02 ± 0.11 in visceral fat). The mean CpG methylation level at CGi1 and CGi2 was found to be low (2–6% and 1–4%, respectively; Fig. 3) in the samples analyzed. In subcutaneous fat, DNA methylation was significantly higher in ASE samples for two CpG sites within CGi2 at genomic positions in chromosome 8:18525215 (*p* = 0.030) and 8:18525237 (*p* = 0.031). In visceral fat, the CpG site at the position 8:18527678 was significantly higher methylated in ASE samples than in a control group (*p* = 0.017) (Fig. 3). For intragenic CGi3, the mean DNA methylation was high (67–97%) in both fat deposits, but did not differ statistically between ASE and control samples (Additional file 5).

## Discussion

Allelic imbalance in genes involved in regulating porcine lipid metabolism and fat tissue physiology has not so far been widely investigated, and there are only a few reports that relate transcriptome based allele specific expression to lipid metabolism or fatness traits. In a study where *l. dorsi* muscles were analyzed during prenatal development, a total of 11,300 variants showed allelic imbalance [20]. Of these, 3 SNPs in *SCD*, *NR3C1* and *PGM1* were associated with porcine fatness and growth traits. Esteve-Codina et al. [21] investigated testicular transcriptome in two pigs with extremely divergent phenotypes including fatness traits. The lipid metabolism category was overrepresented by differentially expressed genes but ASE was detected for only 4% of transcripts. On the other hand, Schachtschneider et al. [22] reported that of eight porcine tissues tested, fat showed the greatest number of ASE genes (225) and muscle the least (135). These studies analyzed single animals and the ASE results were not validated by an alternative method. RNA-seq technology can detect genes with imbalanced allelic expression but may produce false positive ASE hits [23]. Allelic expression imbalance has also been analyzed for several genes thought to affect pig meat quality and fatness traits. Deviations between allelic transcript levels were detected for *ADRB2* in *l. dorsi* muscle [24], *SERPINA6* and *APOA2* in liver [25, 26] but not for *ADIPOQ* in skeletal muscles and backfat [27]. Our study analyzed ASE of four functional candidate genes encoding transcription factors (*PPARA, PPARG, SREBF1*) and a coactivator of multiple transcription factors (*PPARGC1A*) using pyrosequencing as an accurate and sensitive means of quantifying allelic transcript proportions [28].

We demonstrated that both alleles of *PPARG* and *SREBF1* are expressed at similar levels in subcutaneous and visceral fat and *l. dorsi* muscle in 30 unrelated animals representing three commercial pig breeds. Interestingly, recent studies of human *PPARG* revealed allele-specific expression in adipose tissue and led to the identification of a regulatory variant rs4684847 that is associated with risk of type 2 diabetes [29, 30].

Although we detected single PLW individuals showing ASE of *PPARA* in subcutaneous and visceral fat, the mean allelic transcript ratios between cDNA and genomic DNA did not differ significantly, probably due to the small number of individual samples analyzed. We did not detect any other exonic SNP in *PPARA* that could serve as a reporter SNP in our populations that would have enabled allele discrimination in a larger number of heterozygotes, a common limiting factor in such studies. Because *PPARA* is an interesting candidate gene associated with numerous porcine fatness traits [31, 32], further investigation of possible *cis*-regulatory elements affecting its expression is recommended.

Differential allelic expression occurred most frequently for *PPARGC1A*, and bi-directional ASE (A or T allele overrepresented) was detected in fat deposits of PL, PLW and Pietrain pigs. Of the tissues analyzed, we found the greatest number of samples showing allelic imbalance in visceral fat. Tissue and site-specific regulation and function of *PPARGC1A* have been previously highlighted by the finding that its expression level depends on the anatomical location (3rd–4th rib or near the 4th lumbar vertebra) within the porcine *l. dorsi* muscle [33].

The *PPARGC1A* gene encodes a versatile coactivator of many nuclear receptor families, including PPARs (peroxisome proliferator activated receptors) and is involved in stimulation of mitochondrial biogenesis, regulation of glucose, fatty-acid metabolism and muscle fiber type formation [34]. *PPARGC1A* is thus a promising candidate gene for fatness and meat quality traits in livestock, and there have been several reports of associations of its polymorphism or expression with relevant pig production traits [33, 35–38]. Ours is the first study to show ASE of *PPARGC1A* in mammalian tissues.

ASE may be a useful means of identifying and dissecting the genetic or epigenetic factors responsible for differences in transcript expression. A search for potential *cis*-acting regulatory variants using quantification of allelic proportions has successfully identified a variant affecting expression of human *IL13* [19]. Based on association between environment and ASE some risk factors for complex diseases were identified in humans. For example, increased allelic imbalance of *VNN1* in whole blood was associated with elevated BMI [39]. In another study, allele-specific expression of several genes in islets (*ANPEP*, *CAMK2B*, *HMG20A*, *KCNJ11*, *NOTCH2*, *SLC30A8* and *WFS1*) showed that even subtle changes of gene dosage may have significant consequences for development of type 2 diabetes [40]. These results indicate that analysis of ASE is useful to understand pathophysiology of human diseases. It can be anticipated that application of this approach in livestock studies will allow identify molecular background of complex traits, including fatness.

Here, we attempted to decipher potential regulatory elements in *PPARGC1A* but the promoter SNPs identified (rs337351686, rs340650517, rs336405906 and rs345224049) did not affect allelic transcript ratios. An earlier study reported that genes displaying bi-directional ASE show greater variability in methylation of promoter CpG sites than genes with one-directional ASE [41]. Thus, we analyzed methylation at CpG pairs in the 5′-flanking region as well as in exon 6 of *PPARGC1A*. The role of gene body methylation as an epigenetic mark is not clear, but it is known that exons are more highly methylated than intergenic regions, and the gene body methylation level is positively correlated with gene expression [22, 42]. We compared methylation status between samples showing similar and imbalanced expression of two *PPARGC1A* alleles for two CpG islands in the 5′-flanking region. We found subtle but statistically significant differences in methylation level at two CpG sites in subcutaneous fat and one CpG in visceral fat. Although small, these differences may potentially contribute to variation in allelic expression because they may indicate expression changes in particular cell types. The importance of CpG methylation within the *PPARGC1A* promoter to adiposity has been highlighted by the finding that its methylation status in early childhood can predict body fat percent in older children [43].

The bi-directional nature of ASE indicates that regulatory elements affecting *PPARGC1A* are not in linkage disequilibrium with the exonic rSNP (rs45430917) used to quantify allele proportions. Our results showed no evidence that promoter SNPs that were commonly distributed in PLW and PL heterozygotes for rSNP affected *PPARGC1A* allelic expression. We suggest that the *PPARGC1A* may be subject to complex regulation, probably by long-range regulatory factors such as genetic variants or *cis*-regulatory chromatin modifications but CpG methylation may be to some extent involved. Future studies are necessary to identify functional *cis*-acting elements that affect *PPARGC1A* allelic expression in pig tissues.

## Conclusions

Ours is the first study of allele-specific expression of candidate genes involved in regulation of adipogenesis and lipid metabolism in subcutaneous fat, visceral fat and *l. dorsi* muscle of several pig breeds. *PPARG* and *SREBF1* allele expression was balanced. Some imbalance of allelic transcripts was found for *PPARA,* but the greatest prevalence of ASE was detected for *PPARGC1A* in visceral fat of Polish Large White and Polish Landrace pigs. The bi-directional character of ASE of *PPARGC1A* shows that underlying *cis*-regulatory elements are not in linkage disequilibrium with the SNP used to measure allelic proportions. We detected small differences of DNA methylation levels within CpG islands that can be associated with *PPARGC1A* allelic expression in subcutaneous and visceral fat. Further studies are necessary to identify other regulatory elements localized outside the proximal promoter region of *PPARGC1A*.

## Methods

### Animals and tissue collection

A total of 145 female pigs representing Polish Large White (PLW; *n* = 51), Polish Landrace (PL; *n* = 35), Duroc (*n* = 38) and Pietrain (*n* = 21) breeds were analyzed. Gilts were kept under identical environmental conditions, fed ad libitum with the same commercial mix fodder, slaughtered at 100 kg (SD = 1.8) weight and dissected at the local Pig Testing Station (Pawlowice, Poland). Peripheral blood, *longissimus dorsi* (*l. dorsi*) muscle, subcutaneous and visceral fat tissues were collected. Tissue samples were snap frozen in liquid nitrogen and stored at -80 °C.

### Genotyping

Genomic DNA for genotyping was isolated from peripheral blood of 145 pigs using Blood Mini kit (A&A Biotechnology). PCR reactions were performed using primers overlapping known exonic SNPs that were used as reporter SNPs (rSNP) to quantify allele-specific expression: rs342258309 (A > G) in *PPARA,* rs319172675 (A > G) in *PPARG*, rs45430917 (A > T) in *PPARGC1A*, and rs712230598 (C > T) in *SREBF1*. For *SREBF1*, genotypes of some pigs were retrieved from our previous study [44]. Sequences of PCR primers are shown in Additional file 6. Prior to Sanger sequencing amplicons were purified using Exonuclease I (Thermo Scientific) and Alkaline Phosphatase (Thermo Scientific) and sequencing PCR performed using BigDye Terminator v.3 Cycle Sequencing kit (Thermo Scientific). Sequencing products were filtered on Sephadex G-50 (Sigma-Aldrich) and separated by capillary electrophoresis on 3130 Genetic Analyzer (Applied Biosystems).

### Quantification of allele proportions

Quantitative analysis was performed only for breeds where at least 10 heterozygotes were found for each rSNP. Total RNA was extracted from tissue samples using Direct-zol RNA MiniPrep kit (Zymo Research) and TriPure Isolation Reagent (Roche) according to manufacturer’s protocol. Prior to cDNA synthesis, 1 μg RNA was digested with DNase I (Sigma-Aldrich) to remove contaminating genomic DNA. One-strand cDNA synthesis was performed using a Transcriptor High Fidelity cDNA Synthesis Kit (Roche). All cDNA samples were tested for the presence of contaminating genomic DNA by a PCR reaction with primers specific for genomic DNA and cDNA. The cDNA samples were then used to amplify fragments encompassing analyzed SNPs that were used for allele quantification by pyrosequencing. Assays were designed using PyroMark Assay Design 2.0 software (Qiagen). Sequences of primers used for PCR amplifications and pyrosequencing are shown in Additional file 6. Pyrosequencing reactions were performed using PyroMark Q48 Advanced Reagents (Qiagen) and analyzed on Pyromark Q48 Autoprep system (Qiagen). Allelic proportions were first quantified as a percentage of incorporated nucleotides for the tested rSNP using Pyromark Q48 Autoprep 2.4.2 software (Qiagen) and allelic ratios were then calculated by dividing the percentage of one allele by the other. PCR products obtained from genomic DNA (gDNA) and amplified with the same primer pairs as cDNA samples were also pyrosequenced. In gDNA of heterozygotes, an equimolar ratio of both allelic transcripts is expected. Any bias resulting from variations in nucleotide incorporation during pyrosequencing reaction was normalized by dividing the allelic ratio of cDNA and gDNA samples by a mean allelic ratio derived from gDNA for each gene tested [45, 46]. Individual samples were defined as imbalanced when the allelic proportion (mean of two measurements) exceeded a 60:40 threshold [46], i.e. allelic transcript ratios were > 1.5 for one-directional and < 0.667 or > 1.5 for bi-directional ASE. The deviation of mean allelic expression between cDNA and gDNA for each breed was tested by two-tailed *t* test with unequal variances using log_10_-transformed allelic transcript ratios [19, 45]. For *PPARGC1A*, the bi-directional character of ASE was neutralized by dividing the higher percentage by the lower as previously described [46].

### Promoter sequence analysis

To search for polymorphic variants potentially associated with ASE, Sanger sequencing of two amplicons encompassing 1010 bp of the proximal promoter and 5′-untranslated region (5’UTR) of *PPARGC1A* was performed as described above. In addition, rs336405906 (c.-2885G > T) and rs345224049 (c.-2894G > A) SNPs were genotyped as putatively associated with *PPARGC1A* expression according to Kim et al. [16]. Sequences of PCR primers are shown in Additional file 6. MatInspector software (Genomatrix) was used for in silico analysis of putative transcription factor binding sites. Haplotypes were predicted using Haploview (Broad Institute). Association between promoter SNP and allelic transcript expression was performed using a two-tailed *t* test with unequal variances of log_10_-transformed and phase-corrected allelic transcript ratios of heterozygous versus homozygous individuals as described by Forton et al. [19].

### CpG methylation analysis

Two CGi in 5′-flanking region (CGi1 SSC8:18527230–18528335; CGi2 SSC8:18524520–18526314) based on NCBI database and in exon 6 of *PPARGC1A* (CGi3 SSC8:17866933–17867385) based on ENSEMBL database, were selected and primers for CpG methylation analysis (Additional file 6) were designed using PyroMark Assay Design 2.0 software (Qiagen). DNA was purified from subcutaneous and visceral fat by phenol:chloroform:isoamyl alcohol (25:24:1, Sigma-Aldrich) extraction. Methylated and unmethylated controls were prepared with CpG Methyltransferase (Thermo Scientific) and REPLI-g Mini Kit (Qiagen), respectively. Five hundred nanograms DNA was bisulfite-converted using a EZ DNA Methylation-Gold Kit (Zymo Research). PCR reactions were performed on bisulfite-converted DNA using a Pyromark PCR Kit (Qiagen) according to manufactuer’s recommendations. Methylation analysis was carried out by pyrosequencing using Pyromark Q48 Advanced CpG reagents (Qiagen) and analyzed on Pyromark Q48 Autoprep system (Qiagen). CpG methylation level (%) was compared with Student t test between ASE samples (*n* = 5 for subcutaneous fat and *n* = 10 for visceral fat) and control samples (*n* = 10 for subcutaneous and *n* = 10 for visceral fat) with similar expression of both alleles.

## Additional files

Additional file 1: Genotype frequencies of rSNPs in *PPARA, PPARG, PPARGC1A* and *SREBF* genes in tested pig breeds. (DOC 42 kb)

Additional file 2: Distribution of allelic transcript ratios for a) *PPARG*; b) *SREBF1*; c) *PPARA* in tissues and genomic DNA of analyzed breeds. Each boxplot shows the first quartile, median, third quartile and the whiskers show the minimum and maximum allelic transcript ratio values. S. FAT – subcutaneous fat, V. FAT – visceral fat, L. M. – *longissimus dorsi* muscle, gDNA – genomic DNA. (TIF 87 kb)

Additional file 3: SNPs affecting transcription factor (TF) binding to *PPARGC1A* promoter, TF tissue expression and consensus sequences. (DOC 41 kb)

Additional file 4: Genotype frequencies for SNPs in *PPARGC1A* 5′-flanking sequence in heterozygous samples for exonic reporter SNP rs45430917. (DOC 32 kb)

Additional file 5: Mean percentage of 5-methylcytosine (5-mC) ± SD within CGi3, localized in exon 6 of *PPARGC1A* in fat deposits of ASE samples and control groups. The particular cytosines in each fragment analyzed are indicated as CpG1, CpG2, etc. ASE groups included *n* = 5 samples for subcutaneous fat and *n* = 10 samples for visceral fat. Control groups with similar expression of both alleles included *n* = 10 samples for subcutaneous and *n* = 10 samples for visceral fat. (TIF 1950 kb)

Additional file 6: PCR primers used for genotyping, quantification of allelic transcript proportions and CpG methylation analysis. (DOC 46 kb)

## Abbreviations

5-mC: 5-methylcytosine
ASE: Allele-specific expression
BMI: Body mass index
CGi: CpG island
*l. dorsi*: *Longissimus dorsi*
PL: Polish Landrace
PLW: Polish Large White
*PPARA*: Peroxisome proliferator activated receptor alpha
*PPARG*: Peroxisome proliferator activated receptor gamma
*PPARGC1A*: PPARG coactivator 1 alpha
rSNP: Reporter SNP
SD: Standard deviation
SNP: Single nucleotide polymorphism
*SREBF1*: Sterol regulatory element binding transcription factor
SSC8: *Sus scrofa* chromosome 8
UTR: Untranslated region
