# Supplementary material for: Investigation of allele-specific expression of genes involved in adipogenesis and lipid metabolism suggests complex regulatory mechanisms of PPARGC1A expression in porcine fat tissues
Source: BMC Genet. 2018 Nov 29;19:107. doi: 10.1186/s12863-018-0696-6 (PMC6267897; doi:10.1186/s12863-018-0696-6)
Supplement: Supplementary file 1 — Genotype frequencies of rSNPs in PPARA, PPARG, PPARGC1A and SREBF genes in tested pig breeds. (DOC 42 kb) [file 12863_2018_696_MOESM1_ESM.doc]

**Additional file 1.** Genotype frequencies of rSNPs in *PPARA, PPARG, PPARGC1A* and *SREBF* genes in tested pig breeds.

|  | Genotype frequency | | |
| --- | --- | --- | --- |
| Breed^a^ | *PPARA,* rs342258309 | | |
|  | AA | **AG** | GG |
| PLW (n=51) | 0.784 | **0.196** | 0.020 |
| PL (n=35) | 0.771 | **0.200** | 0.029 |
| Duroc (n=38) | 0.421 | **0.395** | 0.184 |
| Pietrain (n=21) | 0.714 | **0.238** | 0.048 |
|  | *PPARG*, rs319172675 | | |
|  | AA | **AG** | GG |
| PLW (n=51) | 0.588 | **0.373** | 0.039 |
| PL (n=35) | 0.514 | **0.457** | 0.029 |
| Duroc (n=38) | 0.447 | **0.553** | 0.000 |
| Pietrain (n=21) | 1.000 | **0.000** | 0.000 |
|  | *PPARGC1A*, rs45430917 | | |
|  | AA | **AT** | TT |
| PLW (n=51) | 0.353 | **0.333** | 0.314 |
| PL (n=35) | 0.429 | **0.429** | 0.142 |
| Duroc (n=38) | 0.079 | **0.368** | 0.553 |
| Pietrain (n=21) | 0.000 | **0.619** | 0.381 |
|  | *SREBF1*, rs712230598 | | |
|  | CC | **CT** | TT |
| PLW (n=51) | 0.020 | **0.039** | 0.941 |
| PL (n=35) | 0.171 | **0.515** | 0.314 |
| Duroc (n=38) | 0.237 | **0.579** | 0.184 |
| Pietrain (n=21) | 0.143 | **0.524** | 0.333 |

^a^PLW – Polish Large White; PL – Polish Landrace
