# Supplementary figures and images for: Investigation of allele-specific expression of genes involved in adipogenesis and lipid metabolism suggests complex regulatory mechanisms of PPARGC1A expression in porcine fat tissues
Source: BMC Genet. 2018 Nov 29;19:107. doi: 10.1186/s12863-018-0696-6 (PMC6267897; doi:10.1186/s12863-018-0696-6)

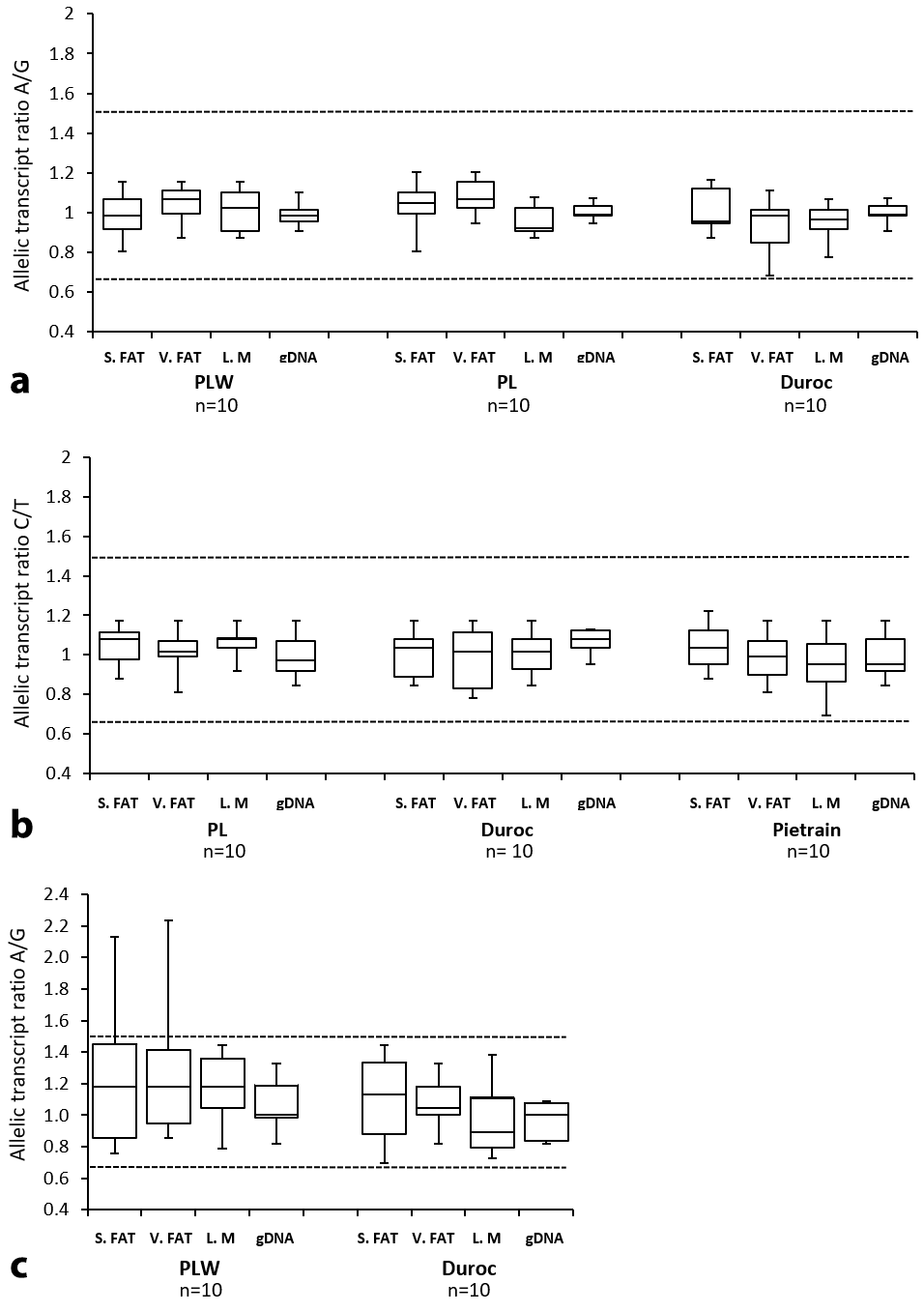

Supplement: Supplementary file 2 — Distribution of allelic transcript ratios for a) PPARG; b) SREBF1; c) PPARA in tissues and genomic DNA of analyzed breeds. Each boxplot shows the first quartile, median, third quartile and the whiskers show the minimum and maximum allelic transcript ratio values. S. FAT – subcutaneous fat, V. FAT – visceral fat, L. M. – longissimus dorsi muscle, gDNA – genomic DNA. (TIF 87 kb) [file 12863_2018_696_MOESM2_ESM.tif]

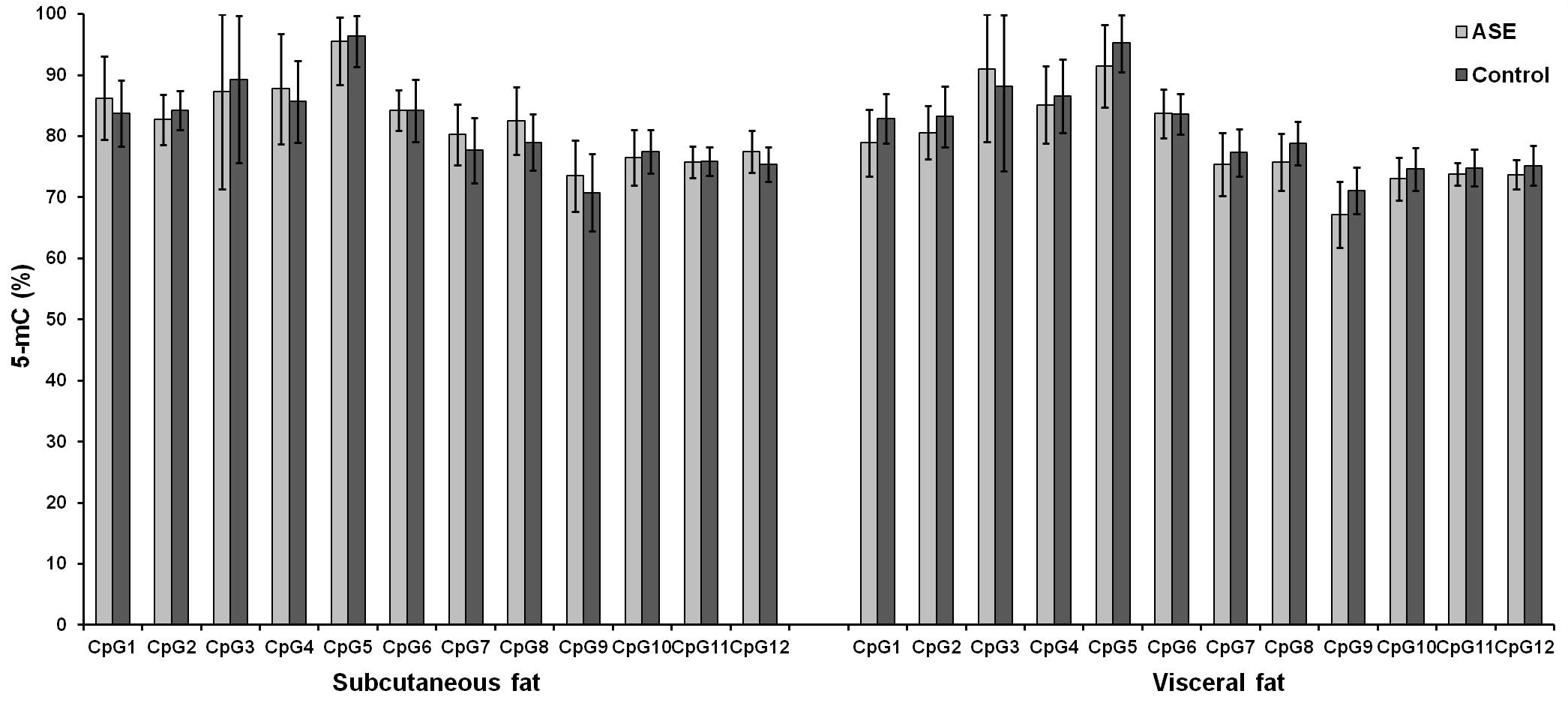

Supplement: Supplementary file 5 — Mean percentage of 5-methylcytosine (5-mC) ± SD within CGi3, localized in exon 6 of PPARGC1A in fat deposits of ASE samples and control groups. The particular cytosines in each fragment analyzed are indicated as CpG1, CpG2, etc. ASE groups included n = 5 samples for subcutaneous fat and n = 10 samples for visceral fat. Control groups with similar expression of both alleles included n = 10 samples for subcutaneous and n = 10 samples for visceral fat. (TIF 1950 kb) [file 12863_2018_696_MOESM5_ESM.tif]
