# Supplementary material for: Investigation of allele-specific expression of genes involved in adipogenesis and lipid metabolism suggests complex regulatory mechanisms of PPARGC1A expression in porcine fat tissues
Source: BMC Genet. 2018 Nov 29;19:107. doi: 10.1186/s12863-018-0696-6 (PMC6267897; doi:10.1186/s12863-018-0696-6)
Supplement: Supplementary file 3 — SNPs affecting transcription factor (TF) binding to PPARGC1A promoter, TF tissue expression and consensus sequences. (DOC 41 kb) [file 12863_2018_696_MOESM3_ESM.doc]

**Additional file 3.** SNPs affecting transcription factor (TF) binding to *PPARGC1A* promoter, TF tissue expression and consensus sequences.

| **SNP** | **TF** | **TF protein expression^1,2,3,4^** | | **TF binding sequence^b^** | **References^c^** |
| --- | --- | --- | --- | --- | --- |
|  |  | **Adipose tissue^a^** | **Skeletal muscle^a^** |  |  |
| rs331429264 (c.-393G>C) | ATF2 | + | + | gagccctgacG/Cgtataataac | 5, 6 |
| rs337351686 (c.-530G>A) | STAT5A | + | + | catgttcC/Tgagatatccaa | 7, 8, 9 |
|  | STAT5B | + | + | ggatatctcA/Ggaacatgag | 7, 10, 11 |
| rs340650517 (c.-644G>A) | KLF4 | + | - | gaaaaagaaaaG/Agcatgcc | 12 |
|  | TP53 | + | + | aagcagagaacggggcatgcC/Ttttt | 13 |

^a^ ‘+’ present; ‘-‘ absent

^b^ Polymorphic sites marked with capital letters

^c^ Reports showing role of TF as regulators of adipogenesis and lipid metabolism

**References**

1. Uhlén M. et al., 2015. Tissue-based map of the human proteome. *Science* 347(6220):1260419, Human Protein Atlas available from [www.proteinatlas.org](https://www.proteinatlas.org/).

2. Vestergaard P.F. et al., 2014. GH signaling in skeletal muscle and adipose tissue in healthy human subjects: impact of gender and age. *Eur J Endocrinol*. 171(5):623-31.

3. Klover P. et al., 2009. Skeletal muscle growth and fiber composition in mice are regulated through the transcription factors STAT5a/b: linking growth hormone to the androgen receptor. *FASEB J.* 23(9):3140-8.

4. Kung C.P. and Murphy M.E., 2016. The role of the p53 tumor suppressor in metabolism and diabetes. *J Endocrinol.* 231(2):R61-R75.

5. Maekawa T. et al., 2010. The role of ATF-2 family transcription factors in adipocyte differentiation: antiobesity effects of p38 inhibitors*. Mol Cell Bio*l. 30(3):613-25.

6. Yao L. et al., 2017. Cold-Inducible SIRT6 Regulates Thermogenesis of Brown and Beige Fat. *Cell Rep*. 20(3):641-654.

7. Wakao H. et al., 2011. Constitutively active Stat5A and Stat5B promote adipogenesis. *Environ Health Prev Med*. 16(4):247-52.

8. Stewart W.C. et al., 2011. STAT5A expression in Swiss 3T3cells promotes adipogenesis in vivo in an athymic mice model system. *Obesity (Silver Spring)* 19(9):1731-4.

9. Dubois S.G. et al. 2006. Decreased expression of adipogenic genes in obese subjects with type 2 diabetes. *Obesity (Silver Spring)* 14(9):1543-52.

10. Gao P. et al., 2015. Signal transducer and activator of transcription 5B (STAT5B) modulates adipocyte differentiation via MOF. *Cell Signal.* 27(12):2434-43.

11. Shipley J.M. and Waxman D.J. et al., 2004. Simultaneous, bidirectional inhibitory crosstalk between PPAR and STAT5b. *Toxicol Appl Pharmacol.* 199(3):275-84.

12. Eisenstein A. et al., 2014. An adenosine receptor-Krüppel-like factor 4 protein axis inhibits adipogenesis. *J Biol Chem*. 289(30):21071-81.

13. Wang Y. et al., 2017. Dynamic transcriptome and DNA methylome analyses on longissimus dorsi to identify genes underlying intramuscular fat content in pigs*. BMC Genomics*. 18(1):780.
