# Supplementary material for: Investigation of allele-specific expression of genes involved in adipogenesis and lipid metabolism suggests complex regulatory mechanisms of PPARGC1A expression in porcine fat tissues
Source: BMC Genet. 2018 Nov 29;19:107. doi: 10.1186/s12863-018-0696-6 (PMC6267897; doi:10.1186/s12863-018-0696-6)
Supplement: Supplementary file 4 — Genotype frequencies for SNPs in PPARGC1A 5′-flanking sequence in heterozygous samples for exonic reporter SNP rs45430917. (DOC 32 kb) [file 12863_2018_696_MOESM4_ESM.doc]

**Additional file 4.** Genotype frequencies for SNPs in *PPARGC1A* 5’-flanking sequence in heterozygous samples for exonic reporter SNP rs45430917.

| Breed^a^ | rs331429264  (c.-393G>C) | | | rs337351686  (c.-530G>A) | | | rs318575008  (c.-531C>G) | | rs340650517  (c.-644G>A) | | rs336405906  (c.-2885G>T) | | rs345224049  (c.-2894G>A) | |
| --- | --- | --- | --- | --- | --- | --- | --- | --- | --- | --- | --- | --- | --- | --- |
|  | GG | CG | | GG | AG | CC | | CG | GG | AG | GG | GT | GG | AG |
| PLW (n=17) | 1.00 | | 0.00 | **0.41**  **(n=7)** | **0.59**  **(n=10)** | 0.82 | | 0.18 | **0.41**  **(n=7)** | **0.59**  **(n=10)** | **0.41**  **(n=7)** | **0.59**  **(n=10)** | 0.94 | 0.06 |
| PL (n=15) | 0.87 | | 0.13 | 0.93 | 0.07 | 1.00 | | 0.00 | 0.93 | 0.07 | 0.93 | 0.07 | **0.60**  **(n=9)** | **0.40**  **(n=6)** |
| Duroc (n=14) | 1.00 | | 0.00 | 1.00 | 0.00 | 1.00 | | 0.00 | 1.00 | 0.00 | 1.00 | 0.00 | 0.79 | 0.21 |
| Pietrain (n=13) | 0.69 | | 0.31 | 1.00 | 0.00 | 1.00 | | 0.00 | 1.00 | 0.00 | 1.00 | 0.00 | 0.31 | 0.69 |

^a^ The groups used for an association analysis of promoter SNPs with allelic transcript ratios are marked with a bold font and numbers of animals in these group is shown in parentheses.
