# Supplementary material for: Investigation of allele-specific expression of genes involved in adipogenesis and lipid metabolism suggests complex regulatory mechanisms of PPARGC1A expression in porcine fat tissues
Source: BMC Genet. 2018 Nov 29;19:107. doi: 10.1186/s12863-018-0696-6 (PMC6267897; doi:10.1186/s12863-018-0696-6)
Supplement: Supplementary file 6 — PCR primers used for genotyping, quantification of allelic transcript proportions and CpG methylation analysis. (DOC 46 kb) [file 12863_2018_696_MOESM6_ESM.doc]

**Additional file 6.** PCR primers used for genotyping, quantification of allelic transcript proportions and CpG methylation analysis.

| **Gene** | **Primer sequences** | **Annealing temperature (^o^C)** | | | | **PCR product length (bp)** |
| --- | --- | --- | --- | --- | --- | --- |
| **Genotyping (Sanger sequencing)** | | | | | | |
| *PPARA* (rs342258309) | F: 5’ ACCCGCCTTTGCACCTTGGACACC  R: 5’ GCGACGTGGCTT TCTGGGACCTTC | 60 | | | | 605 |
| *PPARG* (rs319172675) | F: 5’ GACCACTCCCACTCCTTTGA  R: 5’ AAGAGGGGGAGGAAAGATGA | 60 | | | | 367 |
| *PPARGC1A* (rs45430917) | F: 5’ CCACCCACCACTCCTCCTCATAAA  R: 5’ TTGTCTGCTTCGTCGTCAAAAACA | 60 | | | | 554 |
| *SREBF1* (rs712230598) | F: 5’ TGCACACCCAGGTTCAAAGT  R: 5’ GCGCCTCCATGAAGCTTAG | 57 | | | | 716 |
| **Control of gDNA contamination in cDNA samples** | | | | | | |
| *ACTB* | F: 5’ GGACTTCGAGCAGGAGATGG  R: 5’ GCACCGTGTTGGCGTAGAGG | | 61 | | | gDNA: 329  cDNA: 234 |
| **ASE analysis (pyrosequencing)^1^** | | | | | | |
| *PPARA* (rs342258309) | F: 5’ ACGCAGTGGTTCCATTTCAGTTT  R: 5’ AGCAACAGGCTTTCCCGTATTAG  S: 5’ TTTCAGTTTGCCATTTT | 60 | | | | 65 |
| *PPARG* (rs319172675) | F: 5’ TTTCCACTCCACACTATGAAGACA  R: 5’ TGGAGCTTCAGGTCGTACTTATAA  S: 5’ CCGAGAGCTGATCCA | 60 | | | | 82 |
| *PPARGC1A* (rs45430917) | F: 5’ TGACTGGCAGAGGCAGATGTG  R: 5’ TCCGACGTCTCGGTCAGGTAG  S: 5’ CCACAGACTCAGACCAG | 60 | | | | 66 |
| *SREBF1* (rs712230598) | F: 5’ ACGCTACCGCTCCTCCAT  R: 5’ CCACCACCAGGTCCTTGAG  S: 5’ CTACCGCTCCTCCAT | 60 | | | | 56 |
| **Sequencing of *PPARGC1A* 5’-flanking region** | | | | | | |
| Promoter | F: 5’ GCAGCCGGGAAACTAAGCT  R: 5’ ACACTTGGACTTTTGGAGGCT | | | 60 | | 650 |
| Promoter/5’UTR | F: 5’ TTAAGCGTTACTTCACTGAGGCA  R: 5’ CAATTGCATCGGGAGGGAAG | | | 58 | | 370 |
| Genotyping of rs345224049 and rs336405906 | F: 5’ TGGAACATTATGGGCACTCA  R: 5’ TCCCTCTCTGCCTTGACTGT | | | 56 | | 415 |
| **CpG methylation analysis in *PPARGC1A* (pyrosequencing)^1^** | | | | | | |
| CGi1 | F: 5’ GGTGTGAGTTTGTTTGGGGAGTTTA  R: 5’ TCACCACCCACCCACCTA  S: 5’ TTGTTTGGGGAGTTTAT | | | | 56 | 234 |
| CGi2 | F: 5’ TGGGTGTTTTGGAGAAAAGT  R: 5’ AAAACCCCTCTATAAATAAAAACTTACAA  S: 5’ GTGTTTTGGAGAAAAGTT | | | | 56 | 127 |
| CGi3 | F: 5’ AGTATTGGTTTGAGTTTGTGGAAGAATAT  R: 5’ AAACCCCCTTACAAAACTATAATACCTCC  S: 5’ ATTGATAATAGTTATGGTTATTAAA | | | | 56 | 349 |

^1^ R primers labeled at 5’-end with biotin
